# Supplementary material for: Differentiation of Classical Swine Fever Virus Virulent and Vaccine Strains by CRISPR/Cas13a
Source: Microbiol Spectr. 2022 Sep 29;10(5):e00891-22. doi: 10.1128/spectrum.00891-22 (PMC9603908; doi:10.1128/spectrum.00891-22)
Supplement: Supplemental file 1 — Supplemental material. Download spectrum.00891-22-s0001.pdf, PDF file, 0.6 MB [file spectrum.00891-22-s0001.pdf]

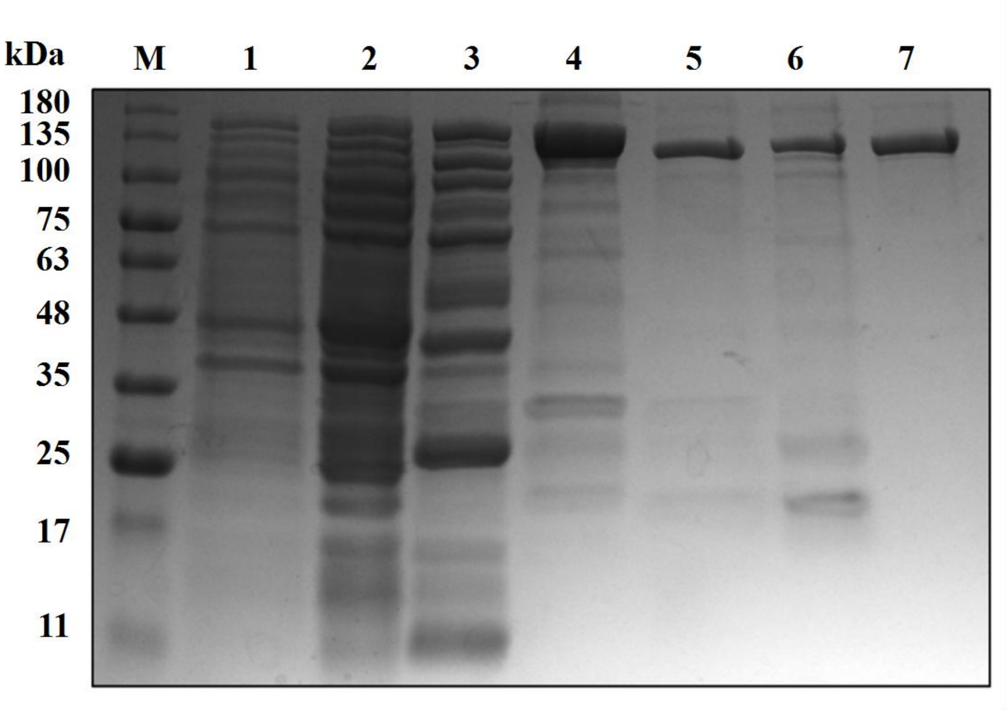

Figure S1 SDS-PAGE of LwaCas13a purification. Supernatant of lysed bacterial LB culture for Ni affinity chromatography. After wased with lysis buffer containing 20 mmol/L imidazole LwaCas13a was eluted with lysis buffer containing 100 mmol/L imidazole. After SUMO protease digestion, LwaCas13a was further purified by SP cation exchange chromatography. M: Colormixed 180 protein marker (Solarbio, China); 1: supernatant of cell lysate; 2: flow-through of Ni sepharose column; 3: 20mM imidazole elution of Ni sepharose column; 4: 100mM imidazole elution of Ni sepharose column; 5: SUMO protease digested production; 6: flow-through of SP column; 7: elution of SP column (purified LwCas13a).

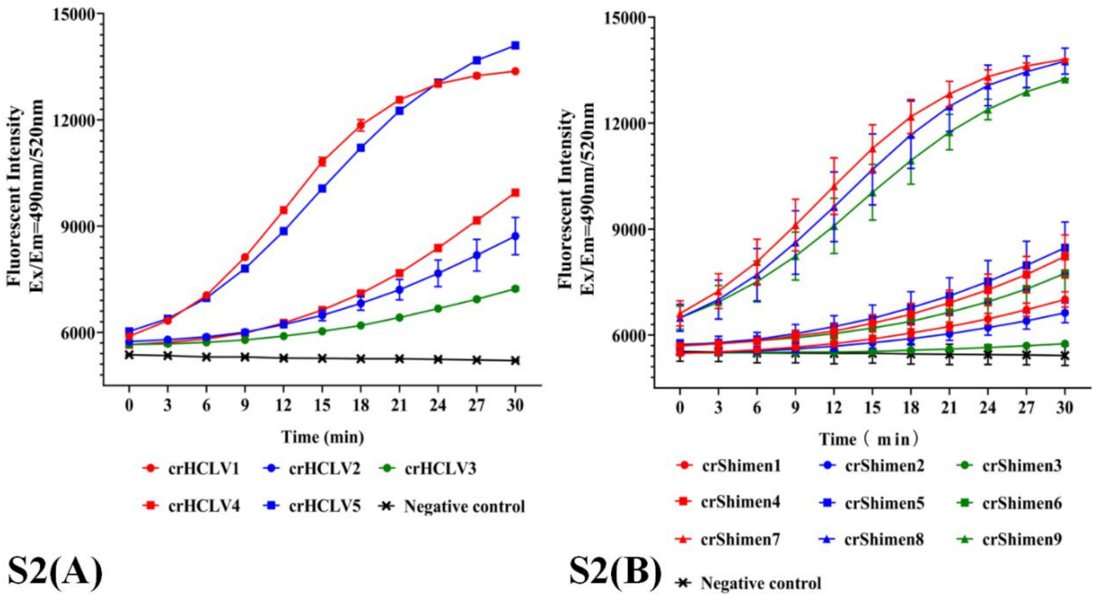

Figure S2 CSFV candidate crRNAs screening by LwaCas13a collateral cleavage assay

(A) Screening of candidate crRNAs for vaccine HCLV strain. Fluorescent kinetics of five candidate crRNAs for HCLV strain were indicated as figure annotation. Negative control used DEPC water to replace crRNA, with other conditions of collateral cleavage assay the same as experimental groups.

(B) Screening of candidate crRNAs for virulent Shimen strain. Fluorescent kinetics of nine candidate crRNAs for Shimen strain were indicated as figure annotation. Negative control used DEPC water to replace crRNA, with other conditions of collateral cleavage assay the same as experimental groups.

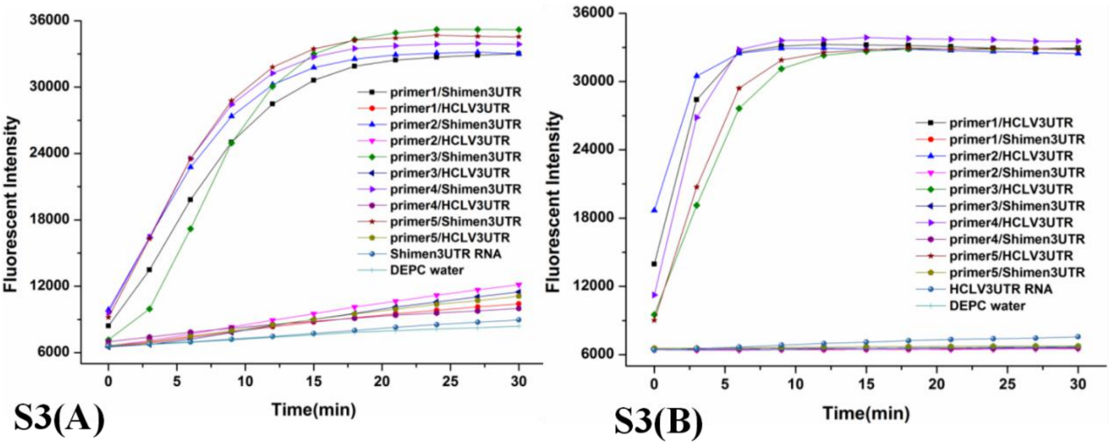

Figure S2. Design and screening of candidate CSFV RAA primers.

(A) RAA primer screening with CRISPR/Cas13a collateral cleavage assay with crShimen7; (B) RAA primer screening with CRISPR/Cas13a collateral cleavage assay with crHCLV5.

CSFV universal RT-RAA primers for both Shimen and HCLV were designed by NCBI Primer-BLAST with parameters set as following: primer length (30-35 nt), primer melting temperatures (54-67°C), amplicon size (100-140 bp) and GC content (40-60%). 24 primer pairs were given by NCBI Primer-BLAST with designated parameters, among which amplification products of 5 primer pairs contain complementary detection region of both crShimen3UTR-7 and crHCLV3UTR-5 and thus were chosen as candidate RT-RAA primers **(Table S5)**.

The RT-RPA products of each primer pairs for both Shimen 3'UTR RNA and HCLV 3'UTR RNA were tested with either crShimen3UTR-7 or crHCLV3UTR-5. Both crShimen7 and crHCLV5 can discriminate the RT-RPA products of Shimen 3'UTR RNA and HCLV 3'UTR RNA with correspondent crRNAs **(Fig. S3A, B)**. There was nearly no difference among these 5 primer pairs in amplifying and detecting CSFV 3'UTR RNAs. In this study, primer pair RPA3UTR-F3/R3 was chosen for RT-RAA.

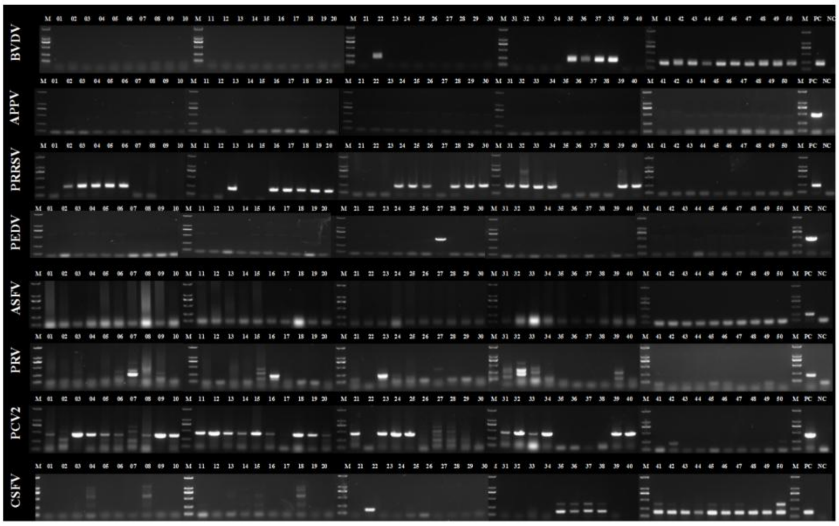
BVDV
APPV
PRRSV
PEDV
ASFV
PRV
PCV2
CSFV
M
PC
NC

S4(A)

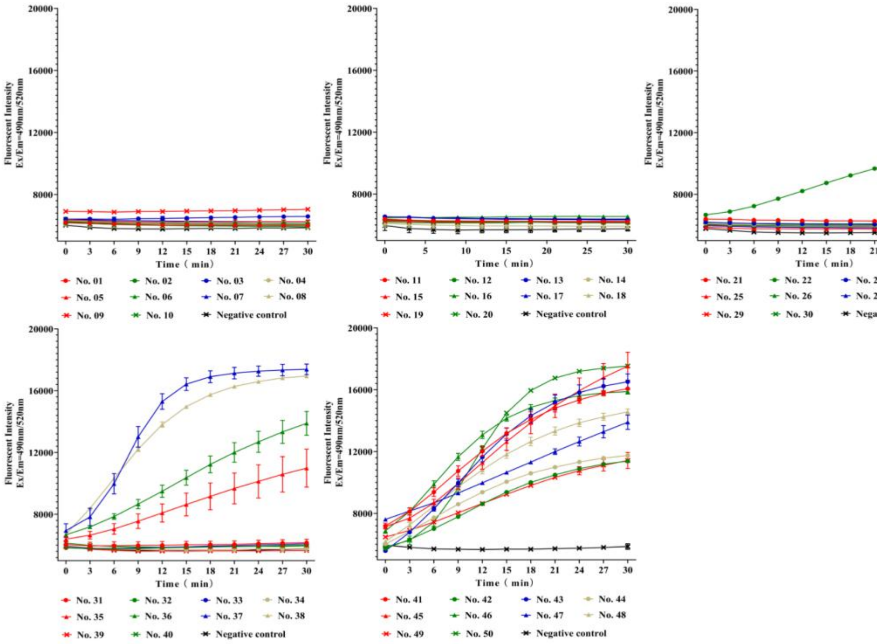
Fluorescent Intensity Ex/Em=490nm/520nm
Time（min）
No. 01
No. 02
No. 03
No. 04
No. 05
No. 06
No. 07
No. 08
No. 09
No. 10
No. 11
No. 12
No. 13
No. 14
No. 15
No. 16
No. 17
No. 18
No. 19
No. 20
No. 21
No. 22
No. 23
No. 24
No. 25
No. 26
No. 27
No. 28
No. 29
No. 30
No. 31
No. 32
No. 33
No. 34
No. 35
No. 36
No. 37
No. 38
No. 39
No. 40
No. 41
No. 42
No. 43
No. 44
No. 45
No. 46
No. 47
No. 48
No. 49
No. 50
Negative control

S4(B)

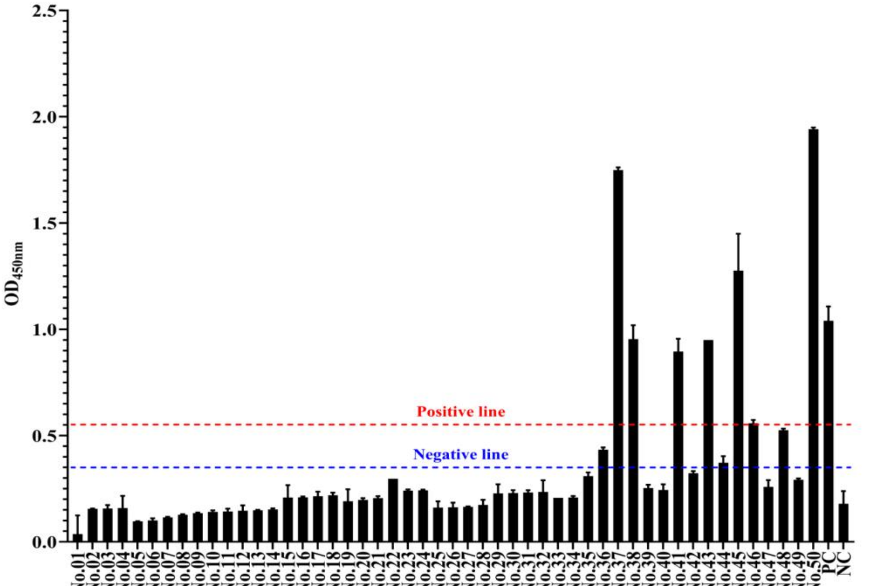
OD450nm
Positive line
Negative line
PC
NC

S4(C)

Figure S4. Comparison of nPCR, HUDSON-RT-RAA-CRISPR/Cas13a and antigen ELISA for testing 50 spleen tissue samples with suspected signs

(A) Agarose gel electrophoresis of PCR for 50 spleen samples. PCR detection for each porcine virus were indicated on the left of each gel, while sample numbers of each spleen sample were indicated above each gel. M: Tans2K DNA marker (TransGege, China); PC, positive control, cDNA of vial cell cultures used in specificity experiment, except for ASFV that was used certified reference genomic nucleic acids as template. NC, negative control, DEPC water.

(B) HUDSON-RT-RAA-CRISPR/Cas13a detection for 50 spleen samples. Fluorescent kinetics in detecting each sample were indicated as figure annotation. Negative control used DEPC water to replace supernatant of tissue honogenates, with other conditions of HUDSON treatment, RT-RAA and collateral cleavage assay the same as experimental groups.

(C) CSFV antigen ELISA detection for 50 spleen samples. Sample numbers of each spleen sample were indicated at horizontal axis. PC and NC: positive control and negative control provided by ELISA kit to determine positive line, negative line and suspected section.

Table S1 CSFV reference strains used in this study

Strain	Region	Genotype	Accession No.
HCLV	China	1.1	AF091507
Shimen	China	1.1	AF092448
Bresciax90	Netherland	1.2	M31768
CSF1058	Cuba	1.4	KX576461
SXCDK	China	2.1a	GQ923951
SXYL2006	China	2.1b	GQ122383
HNSD-2012	China	2.1c	JX218094
JSZL	China	2.1d	KT119352
GD19	China	2.1g	KU504339.1
Penevezys	Lithuania	2.1h	HQ148063.1
GD317/2011	China	2.1i	KY132096.1
39	China	2.2	AF407339
Jambul	Bulgaria	2.3	HQ148062
JJ9811	Korea	3.2	KF669877
TW-94	China	3.4	AY646427

Table S2 Viruses used in this study

Virus	Strain	Viral load	Source
CSFV	Shimen	3.5×10^4 copies/μL	PK15 cell culture
CSFV	HCLV	1.8×10^3 copies/μL	ST cell culture
BVDV	AV69	6.4×10^4 copies/μL	MDBK cell culture
APPV	HeLY/2017	—	Positive cDNA
PRRSV	HN07-1	7.8×10^4 copies/μL	MARC 145 cell culture
PEDV	Hubei2016	5.0×10^5 copies/μL	VERO cell culture
ASFV	Genotype Ⅱ Genomic DNA	1.4×10^4 copies/μL	National Centified Refference Materials of China
PRV	HeNLH/2017	8.3×10^5 copies/μL	PK15 cell culture
PCV 2	HN-LB-2016	2.3×10^8 copies/μL	PK15 cell culture

Table S3 crRNAs used in this study

crRNAs	sequences
crShimen1	GAUUUAGACUACCCCAAAAACGAAGGGGACUAAAACAAAAAAUUAGUGUUAUCUACAAUAGGGU
crShimen2	GAUUUAGACUACCCCAAAAACGAAGGGGACUAAAACUAAAAAAUUAGUGUUAUCUACAAUAGGG
crShimen3	GAUUUAGACUACCCCAAAAACGAAGGGGACUAAAACAUAAAAAAUUAGUGUUAUCUACAAUAGG
crShimen4	GAUUUAGACUACCCCAAAAACGAAGGGGACUAAAACAAUAAAAAAUUAGUGUUAUCUACAAUAG
crShimen5	GAUUUAGACUACCCCAAAAACGAAGGGGACUAAAACAAAUAAAAAAUUAGUGUUAUCUACAAUA
crShimen6	GAUUUAGACUACCCCAAAAACGAAGGGGACUAAAACUAAAUAAAAAAUUAGUGUUAUCUACAAU
crShimen7	GAUUUAGACUACCCCAAAAACGAAGGGGACUAAAACAUAAAUAAAAAAUUAGUGUUAUCUACAA
crShimen8	GAUUUAGACUACCCCAAAAACGAAGGGGACUAAAACAAUAAAUAAAAAAUUAGUGUUAUCUACA
crShimen9	GAUUUAGACUACCCCAAAAACGAAGGGGACUAAAACAAAUAAAUAAAAAAUUAGUGUUAUCUAC
crHCLV1	GAUUUAGACUACCCCAAAAACGAAGGGGACUAAAACAUAAAAAAGAAAAAAGAAAAGUAGUGUA
crHCLV2	GAUUUAGACUACCCCAAAAACGAAGGGGACUAAAACAAUAAAAAAGAAAAAAGAAAAGUAGUGU
crHCLV3	GAUUUAGACUACCCCAAAAACGAAGGGGACUAAAACAAAUAAAAAAGAAAAAAGAAAAGUAGUG
crHCLV4	GAUUUAGACUACCCCAAAAACGAAGGGGACUAAAACUAAAUAAAAAAGAAAAAAGAAAAGUAGU
crHCLV5	GAUUUAGACUACCCCAAAAACGAAGGGGACUAAAACAUAAAUAAAAAAGAAAAAAGAAAAGUAG
cr2.17	GAUUUAGACUACCCCAAAAACGAAGGGGACUAAAACAUAAAUAAUAAGUUAGUGUUAUUUACAA

Table S4 Primers and conditions of PCR used in this study

Viruses	Initial PCR		Nested PCR		Reference
	Primers	Conditions	Primers	Conditions	
**BVDV**	Forward:5'-GGCTAGCCATGCCCTTAG-3' Reverse:5'-CCATGTGCCATGTACAG-3'	1 cycle of 95 °C, 5 min. 20 cycles of 95 °C, 30 s; 55 °C, 30 s; 72 °C, 30 s. 1 cycle of 72 °C, 10 min.	Forward:5'-CCTGAGTACAGGGDAGTCGTCA-3' Reverse:5'-CCATGTGCCATGTACAG-3'	1 cycle of 95 °C, 5 min. 20 cycles of 95 °C, 30 s; 50 °C, 30 s; 72 °C, 35 s. 1 cycle of 72 °C, 10 min.	(1)
**APPV**	Forward:5'-ATGTTTCTCAGGGTCGGA-3' Reverse:5'-GGTCATCTTCCGCAGTTA-3'	1 cycle of 94 °C, 5 min. 30 cycles of 94 °C, 1 min; 42 °C, 1 min; 72 °C, 1 min. 1 cycle of 72 °C, 7 min.	Forward:5'-TGTGGAGATGACGGCTTTC-3' Reverse:5'-CGGGATAACTGCGACTGAT-3'	1 cycle of 94 °C, 5 min. 35 cycles of 94 °C, 1 min; 45 °C, 1 min; 72 °C, 1 min. 1 cycle of 72 °C, 7 min..	(2)
**PRRSV**	Forward:5'-TCGTGTTGGGTGGCAGAAAAGC-3' Reverse:5'-GCCATTCACCACACATTCTTCC-3'	1 cycle of 95 °C, 5 min. 40 cycles of 95 °C, 30 s; 58 °C, 30 s; 72 °C, 30 s. 1 cycle of 72 °C, 10 min.	Forward:5'-CCAGATGCTGGGTAAGATCATC-3' Reverse:5'-CAGTGTAACTTATCCTCCCTGA-3'	1 cycle of 95 °C, 5 min. 30 cycles of 95 °C, 30 s; 58 °C, 15 s, 72 °C, 30 s. 1 cycle of 72 °C, 10 min.	(3)
**PEDV**	Forward:5'-AACACTTAGCCTACCACA-3' Reverse:5'-GTGGAATCATTGGACAA-3'	1 cycle of 95 °C, 5 min. 30 cycles of 95 °C, 30 s; 49 °C, 30 s, 72 °C, 30 s. 1 cycle of 72 °C, 10 min.	Forward:5'-GAAAACCAGGGTGTCAA-3' Reverse:5'-GAAATACCATCCTCACCAG-3'	1 cycle of 95 °C, 5 min. 30 cycles of 95 °C, 30 s; 51 °C, 30 s; 72 °C, 30 s; 1 cycle of 72 °C, 10 min.	(4)
**ASFV**	Forward:5'-AGTTATGGGAAACCCGACCC-3' Reverse:5'-CCCTGAATCGGAGCATCCT-3'	1 cycle of 95 °C, 10 min. 40 cycles of 95 °C, 15 s; 62 °C, 30 s; 72 °C, 30 s. 1 cycle of 72 °C, 7 min.			(5)
**PRV**	Forward:5'-ATGGCCATCTCGCGGTGC-3' Reverse:5'-ACTCGCGGTCCTCCAGCA-3'	1 cycle of 95 °C, 10 min. 30 cycles of 95 °C, 30 s; 62 °C, 60 s; 72 °C, 60 s. 1 cycle of 72 °C, 10 min.	Forward:5'-ACGGCACGGGCGTGATC -3' Reverse:5'-GGTTCAGGGTTCACCCGC-3'	1 cycle of 95 °C, 10 min. 30 cycles of 94 °C, 30 s; 62 °C, 30 s; 72 °C, 30 s. 1 cycle of 72 °C, 10 min.	(6)
**PCV 2**	Forward:5'-GGTGGAACTGTACCTTTTTTTGGCCCGC-3' Reverse:5'-CTCCTCCCGCCATACAATCCCCC-3'	1 cycle of 95 °C, 4 min. 35 cycles of 95 °C, 60 s; 58 °C, 60 s; 72 °C, 60 s. 1 cycle of 72 °C, 10 min.	Forward:5'-GAATGGTACTCCTCAACTGCTGTCCCAGC-3' Reverse:5'-CCACTCCCGTTAATTCACACCCAAACC-3'	1 cycle of 95 °C, 4 min. 35 cycles of 95 °C, 60 s; 58 °C, 60 s; 72 °C, 60 s. 1 cycle of 72 °C, 10 min.	(7)

Table S5 Candidate CSFV RAA primer pairs

No.	Sequence	Length/nt	Tm/℃	GC%	Amplicon size/bp
3UTR/RPA-F1	TAATACGACTCACTATAGGGGGGGAACCCGCC AGTAGGACCCTATTGTAGATAA	31	66.75	48.39	133
3UTR/RPA-R1	GAGTGTAGTGTGGTAACTTGAGGTAGTTTGTA	32	64.14	40.62	
3UTR/RPA-F2	TAATACGACTCACTATAGGGGGGAACCCGCCA GTAGGACCCTATTGTAGATAAC	31	66.94	48.39	134
3UTR/RPA-R2	ATGAGTGTAGTGTGGTAACTTGAGGTAGTTTG	32	64.55	40.62	
3UTR/RPA-F3	TAATACGACTCACTATAGGGGGGGAACCCGCC AGTAGGACCCTATTGTAGATA	30	66.56	50.00	124
3UTR/RPA-R3	GTGGTAACTTGAGGTAGTTTGTACCAGTTCTT	32	64.70	40.62	
3UTR/RPA-F4	TAATACGACTCACTATAGGGGGGCCCGCCAGTA GGACCCTATTGTAGATAACA	30	66.75	50.00	127
3UTR/RPA-R4	TGTAGTGTGGTAACTTGAGGTAGTTTGTACCAG	33	65.72	42.42	
3UTR/RPA-F5	TAATACGACTCACTATAGGGGGGACCCGCCAGT AGGACCCTATTGTAGATAAC	30	66.76	50.00	127
3UTR/RPA-R5	GTAGTGTGGTAACTTGAGGTAGTTTGTACCAGT	33	65.72	42.42	

Table S6 Results of nPCR, HUDSON-RT-RAA-CRISPR/Cas13a and ELISA for testing fifty spleen tissues

No	BVDV	APPV	PRRSV	PEDV	ASFV	PRV	PCV2	CSFV		
	PCR								CRISPR	ELISA
01							+			
02			+							
03			+				+			
04			+				+			
05			+				+			
06			+							
07						+				
08							+			
09							+			
10							+			
11							+			
12							+			
13			+				+			
14							+			
15							+			
16			+			+	+			
17			+							
18			+				+			
19			+				+			
20			+				+			
21							+			
22	+							+	+	
23						+	+			
24			+				+			
25			+				+			

(continues)

Table 5 (Continued)

No	BVDV	APPV	PRRSV	PEDV	ASFV	PRV	PCV2	CSFV		
	PCR								CRISPR	ELISA
26			+							
27				+						
28			+							
29			+							
30			+							
31			+				+			
32			+				+			
33			+				+			
34			+				+			
35	+							+	+	
36	+							+	+	±
37	+							+	+	+
38	+							+	+	+
39			+				+			
40			+				+			
41	+							+	+	+
42	+							+	+	
43	+							+	+	+
44	+							+	+	±
45	+							+	+	+
46	+							+	+	±
47	+							+	+	
48	+							+	+	±
49	+							+	+	
50	+							+	+	+

References

1. Givens MD, Galik PK, Riddell KP, Stringfellow DA, Brock KV, Bishop MD, Eilertsen KJ, Loskutoff NM. 2001. Validation of a reverse transcription nested polymerase chain reaction (RT-nPCR) to detect bovine viral diarrhea virus (BVDV) associated with in vitro-derived bovine embryos and co-cultured cells. Theriogenology 56 (5): 787-799.
2. Possatti F, Headley SA, Leme RA, Dall Agnol AM, Zotti E, de Oliveira TES, Alfieri AF, Alfieri AA. 2018. Viruses associated with congenital tremor and high lethality in piglets. Transbound Emerg Dis 65(2): 331-337.
3. Christopher-Hennings JC, Nelson EA, Nelson JK, Hines RJ, Chase CCL. 1995. Detection of porcine reproductive and respiratory syndrome virus in boar semen by PCR. J Clin Microbiol 33 (7): 1730-1734.
4. Shi J, Wang F, Su DP, Xu SF, Luo TX, He DS. 2017. Development and application of a nested RT-PCR method for differentiation between variant and classical PEDV strains. Animal Husbandry ＆ Veterinary Medicine 49 (7): 99-102.
5. Zhang YH, Li QM, Guo JQ, Li DL, Wang L, Wang X, Xing GX, Deng RG, Zhang GP. 2021. An isothermal molecular point of care testing for African swine fever virus using recombinase-aided amplification and lateral flow assay without the need to extract nucleic acids in blood. Front Cell Infect Microbiol 17 (11): 633763.
6. Yoon HA, Eo SK, Aleyas AG, Cha SY, Lee JH, Chae JS, Jang HK, Cho JG, Song HJ. 2006. Investigation of Pseudorabies virus latency in nervous tissues of seropositive pigs exposed to field strain. J Vet Med Sci 68 (2): 143-148.
7. Hamel AL, Lin LL, Sachvie C, Grudeski E, Nayar GP. 2000. PCR detection and characterization of type-2 porcine circovirus. Can J Vet Res 64 (1): 44-52.
